# Proteomics of the temporal cortex in semantic dementia reveals brain-region specific molecular pathology and regulation of the TDP-43-ANXA11 interactome

**DOI:** 10.1186/s40478-025-02077-x

**Published:** 2025-07-25

**Authors:** Suzanne S. M. Miedema, Ana Rajicic, Merel O. Mol, Iryna Paliukhovich, Remco V. Klaassen, Renee van Buuren, Ka Wan Li, Frank T. W. Koopmans, Harro Seelaar, Jeroen G. J. van Rooij, August B. Smit, John C. van Swieten

**Affiliations:** 1 Department of Molecular and Cellular Neurobiology, Center for Neurogenomics and Cognitive Research, Amsterdam Neuroscience, Vrije Universiteit Amsterdam, Amsterdam, The Netherlands; 2 Department of Neurology and Alzheimer Center Erasmus MC, Erasmus University Medical Center, Rotterdam, The Netherlands

**Keywords:** Semantic dementia, FTLD-TDP type C, Temporal cortex, Quantitative proteomics, RNP complex, Presynaptic regulation calcium levels, TDP-43-ANXA11 interactome, Immunohistochemistry

## Abstract

Semantic dementia (SD) is a clinical subtype of frontotemporal dementia characterized by impaired word comprehension and semantic memory, and occurs nearly always sporadically. Neuroimaging typically reveals asymmetric, predominantly left-sided, atrophy of the anterior temporal pole, anterior fusiform gyrus, and the hippocampus. Post-mortem pathological examination shows frontotemporal lobar degeneration TDP type C, characterized by long dystrophic neurites in the temporal cortex and typical round, TDP-43-positive neuronal inclusions in the dentate gyrus. While neuronal loss in the temporal cortex is severe in the end stage of disease, the dentate gyrus seems relatively spared. This characteristic and well-defined disease profile suggests SD patients share a specific underlying disease biology. Recently, we performed the first quantitative proteomic study of the dentate gyrus, uncovering potential SD-specific biological pathways. Here, we report on the first quantitative proteomic study of the temporal cortex in SD. We studied the same patient and non-demented control cohort, enabling comparative analysis between the two brain regions. In addition, we compared our dataset with other frontotemporal lobar degeneration subtypes and Alzheimer’s disease to separate SD disease-specific changes from common neurodegenerative processes. In the temporal cortex, involvement of the ribonucleoprotein complex and presynaptic regulation of cytosolic calcium levels by voltage-gated calcium channels appear unique facets of the SD disease process. Furthermore, we observed a striking difference in the abundance of neuropathological proteins TDP-43 and ANXA11, and their interactors between the temporal cortex and dentate gyrus. The elucidation of these potentially unique disease-specific mechanisms improves our understanding of the pathophysiological processes in SD and paves the way for the discovery of novel therapeutic targets.

**Supplementary Information:**

The online version contains supplementary material available at 10.1186/s40478-025-02077-x.

## Introduction

Semantic dementia (SD), or semantic variant of primary progressive aphasia (svPPA), is a clinical subtype of frontotemporal dementia (FTD) characterized by impaired word comprehension and semantic memory [19, 22, 45]. It has been estimated that the SD subtype constitutes roughly 25–30% of all FTD patients [22]. SD occurs nearly always sporadically [28] and patients show a distinct profile of clinical, neuroimaging, and neuropathological features and a relatively slow disease progression. Neuroimaging typically reveals asymmetric, predominantly left-sided, atrophy of the anterior temporal pole, the anterior fusiform gyrus, and the hippocampus [26, 28, 33, 36]. Post-mortem pathological examination shows specific TDP-43 positive protein aggregates in the brain, a neuropathological entity classified as frontotemporal lobar degeneration (FTLD) TDP type C. This type is characterized by the presence of long dystrophic neurites in the temporal cortex compared to round, neuronal inclusions in the dentate gyrus of the hippocampus [13, 39]. Recently it was shown that another protein, Annexin A11 (ANXA11), co-localizes with TDP-43 to form heteromeric amyloid filaments, which contribute to these inclusions [1]. Furthermore, while neuronal loss in the temporal cortex is severe in the end stage of the disease, subregions of the hippocampus, such as the dentate gyrus seem relatively spared [48]. Despite evidence of volumetric changes in almost all hippocampal regions [8], functions such as episodic memory are relatively preserved in SD patients.

The presence of a characteristic and well-defined disease profile in SD patients suggests they have a shared specific underlying disease biology [29, 46]. As of yet, the pathophysiological mechanisms remain largely unknown and therapeutics are unavailable. Mass spectrometry (MS) based methods of investigating the human proteome have the potential to discover and identify biological pathways and cell types involved in the disease process [7, 9, 30, 32, 35, 38, 44]. Recently [34], we performed the first quantitative proteomic study of SD, in which we examined the dentate gyrus for changes in protein abundance compared to a group of non-demented controls (NDCs). We detected 151 differentially abundant proteins, of which 79 were considered potentially unique to the SD dentate gyrus when compared to other FTLD subtypes and Alzheimer’s disease (AD) proteomes. Functional enrichment indicated pathways related to immune response, metabolic processes, and cell-junction assembly, and we further focused on a cluster of cadherin-catenin complex proteins. These proteins (CTNNB1, JUP, and CDH2) are essential for synaptic signalling in their role at the synaptic membrane and might be uniquely regulated in SD.

One of the great challenges in human proteome studies is the identification of key disease-specific processes, as many of the identified proteome changes are typically observed in many different brain disorders and may represent common pathways of neurodegeneration. Therefore, in-depth comparison across datasets is essential to help differentiate common neurodegenerative alterations from disease specific pathways. Here, we report on the first quantitative proteomic analysis of the post-mortem temporal cortex brain tissue in SD. We studied the same patient and control cohort as for the dentate gyrus, enabling comparative analysis between both brain regions. As these regions show striking differences in SD, a comparison between their proteomes will provide us with insights into region-specific disease changes. In addition, we compared our SD proteome data with published quantitative proteomes of other FTLD subtypes and AD to separate SD disease-specific changes from common neurodegenerative processes. The elucidation of potentially unique disease-specific mechanisms aids in improving our understanding of the pathophysiological processes in SD and paves the way for the discovery of novel therapeutic targets.

## Materials & methods

### Patient tissue collection

Fresh frozen post-mortem cortical brain samples of either the medial, superior, or inferior temporal gyrus were obtained from the Netherlands Brain Bank, Netherlands Institute for Neuroscience, Amsterdam. All selected patients had been clinically followed in the Erasmus Medical Center in Rotterdam. A total of 18 SD patients, with confirmed FTLD-TDP type C pathology [39] and 25 age and sex matched non-demented controls were included (Additional File 1). All patients were previously tested negative for pathogenic germline variants in the major FTD-related genes (*MAPT*, *GRN*, *C9orf72*, *TARDBP*, *TBK1*, *OPTN*, *SQSTM1*, *VCP*, *CHMP2B*, *FUS* [20]).

### Brain tissue preparation and laser capture microdissection

Sections (10 μm) of fresh frozen tissue were mounted on polyethylene naphthalate-membrane slides (Leica, Herborn, DE), fixed in 100% ethanol for 1 min and stained using 1% (wt/vol) Toluidine Blue in H_2_O (Fluka Analytical, Buchs, Switzerland) for 1 min. Laser microdissection was performed using a Leica AS LMD system. Equal volumes of grey matter tissue (1.0 × 10^9^ μm^3^) from the temporal cortical region were collected in Eppendorf tubes containing 30-μL M-PER lysis buffer (Thermo Scientific, Rockford, IL, USA) supplemented with reducing sodium dodecyl sulphate sample buffer (Thermo Scientific). Microdissected tissue was stored at − 80 °C until further use.

### Protein separation by electrophoresis and in‑gel digestion

Microdissected tissue lysates were incubated at 95 °C for 5 min, followed by incubation with 500 mM iodoacetamide for 30 min at room temperature in the dark. Proteins were size separated on a NuPAGE 4–12% Bis–Tris acrylamide gel (Invitrogen, Carlsbad, CA, USA) using MOPS sodium dodecyl sulphate running buffer (Invitrogen) according to the manufacturer’s protocol. Gels were fixed and stained with colloidal Coomassie Blue G-250 while shaking. After destaining in ultrapure H_2_O, each gel lane was cut into blocks of approximately 1 mm^3^ and collected in a 96-wells plate. Destaining, trypsin digestion, and peptide extraction were done as described previously [10].

### Liquid chromatography and mass spectrometry

Each sample of tryptic digest was redissolved in 0.1% formic acid and the peptide concentration was determined by tryptophan-fluorescence assay [52]; 75 ng of peptide was loaded onto an Evotip Pure (Evosep). Peptide samples were separated by standardized 30 samples per day method on the Evosep One liquid chromatography system, using a 15 cm × 150 μm reverse-phase column packed with 1.5 µm C_18_-beads (EV1137 from Evosep) connected to a 20 µm ID ZDV emitter (Bruker Daltonics). Peptides were electro-sprayed into the timsTOF Pro 2 mass spectrometer (Bruker Daltonics) equipped with CaptiveSpray ion source and measured with the following settings: Scan range 100–1700 m/z, ion mobility 0.6 to 1.6 Vs/cm^2^, ramp time 100 ms, accumulation time 100 ms, and collision energy decreasing linearly with the inverse precursor ion mobility from 59 eV at 1.6 Vs/cm^2^ to 20 eV at 0.6 Vs/cm^2^. Operating in dia-PASEF mode, each cycle took 1.8 s and consisted of 1 MS1 full scan and 16 dia-PASEF scans. Each dia-PASEF scan contained two dia-PASEF isolation windows, in total covering 400–1201 m/z (1 Th window overlap) and ion mobility 0.6 to 1.43 Vs/cm^2^. Ion mobility was auto-calibrated at the start of each sample.

### Data-independent acquisition (DIA) data extraction and analysis

DIA-PASEF raw data were processed with DIA-NN 1.8 [14]. An in-silico spectral library was generated from the uniprot human proteome (SwissProt and TrEMBL, canonical sequences only, release 2019–11) using Trypsin/P digestion and at most 1 missed cleavage. Fixed modification was set to carbamidomethylation(C) and variable modifications were oxidation(M) and N-term M excision (at most 1 per peptide). Peptide length was set to 7–30, precursor m/z was limited to 300–1200, both MS1 and MS2 mass accuracy were set to 10 ppm and match-between-runs was enabled. RT-dependent cross-run normalization was enabled with the robust LC quantification strategy. Protein identifiers (isoforms) were used for protein inference. All other settings were left as default.

### Quality control and statistical analysis of differential protein abundance

MS-DAP 1.0.2 [24] was used for downstream analyses of the DIA-NN results. Peptide-level filtering was configured to retain only peptides that were confidently identified in at least N = 3 and at least 75% of samples per sample group. These criteria were applied to all sample groups in the dataset. Peptide abundance values were normalized using the VSN algorithm, followed by protein-level mode-between normalization. Two clear outlier samples (both NDC samples; ID #24 and #25) were identified in the quality control analyses presented in the MS-DAP report and were subsequently excluded from statistical analyses. Differential expression analysis was performed using the MSqRob algorithm [18] and resulting p-values were adjusted for multiple testing using the Benjamini–Hochberg False Discovery Rate (FDR) procedure. Age, sex, and gel number were included as random variable in the regression model. Proteins with differential abundance in SD were defined as having an adjusted *P*-value cut-off of *q* < 0.01.

### Refinement of the SD proteome by comparison to FTLD and AD proteomic changes

To identify proteins potentially unique to SD, we compared our results to previous proteomic studies investigating FTLD and AD pathology. PubMed was searched using the following terms: (‘proteomic*’ OR ‘mass spectrometry’) AND (‘frontotemporal dementia’ OR ‘frontotemporal lobar degeneration’ OR ‘Alzheimer’s disease’). The resulting articles were manually filtered to meet the following criteria: (1) quantitative MS study conducted on brain tissue of FTLD or AD patients compared to non-demented controls; (2) temporal cortex, frontal cortex, entorhinal cortex, or (para)hippocampal tissue; (3) sample size ≥ 5 cases; and (4) full lists of quantified and differentially abundant proteins available. Non-human studies or studies without control group were excluded, as well as studies with overlapping patient cohorts. We extracted lists of all quantified and differentially abundant proteins for comparison to our dataset. Proteins from our SD data set that were never measured in any of the literature (N = 98) were left out from the refinement process (step 0). Detailed information on included studies and our refinement steps can be found in Additional File 2, but shortly summarized: in refinement step (1) we compared our original SD temporal cortex proteome to the AD proteome literature; when our differential protein was measured in ≥ 2 study comparisons and was significantly differentially abundant, either in the same fold change direction or in a mix of fold change directions, the differential protein was filtered out from our list. If it was measured in ≥ 2 study comparisons and significantly differentially abundant but in opposite fold change direction, the protein passed filtering. Also, differential proteins measured in ≥ 2 study comparisons but not significantly differentially abundant or proteins measured in < 2 study comparisons passed filtering. These rules were repeated in refinement step (2) for comparison with FTLD-TAU proteome literature and in refinement step (3) for comparison with FTLD-TDP (with the exception of TDP type c) proteome literature.

### Gene ontology (GO) overrepresentation analyses

Functional GO enrichment analysis was performed using g:Profiler web server (version e111_eg58_p18_30541362) [23] with all settings on default, g:Profiler-based multiple testing correction (g:SCS method) at *q* < 0.05, and with the total set of DIA quantified proteins as background. Separate analyses were run for proteins with higher and lower abundance. For protein groups that matched multiple genes, only the leading gene symbol was used. Classical GO terms, i.e. biological process (BP), cellular component (CC), and molecular function (MF) were examined, taking only terms containing five or more proteins into account. For visualization, only ‘driver’ GO terms are shown. For detailed analysis regarding affected synaptic processes we used SynGO enrichment analysis (version: 20,231,201) [25], with FDR-based multiple testing correction at q < 0.01, and the total set of DIA quantified proteins as background. SynGO analysis was performed on cellular components (CC) and biological processes (BP) ontology terms.

### Cell type enrichment analysis

Cell type enrichment analysis can help to stratify data from mixed cell populations, without the need for physical cell sorting [2, 31]. We recently applied this method on protein expression data for the first time with success [32]. Cell type enrichment analysis of temporal cortical protein signatures in this study was based on a combination of single cell RNAseq (scRNAseq) data of 466 cells from eight adult control donors [12] and single nuclei RNAseq (snRNAseq) data of 15,928 cells from eight adult control donors [21] from temporal cortical tissue of either surgical procedures or post-mortem. Pre-processing and analysis of sc/snRNAseq data sets and subsequent expression-weighted cell type enrichment analysis (EWCE) were done as described previously [32]. In short, for cell type identification of cell clusters in the RNAseq data sets several highly cell-specific proteins were used as markers. Clusters that could not be clearly identified with one cell type were labelled ‘uncharacterized’. For EWCE analysis of our protein signatures, the total set of DIA quantified proteins in this study was used as the background set and significance was set at *q* < 0.05.

### Assessment of the presence of major brain cell type-specific protein populations in proteomic data

We aimed to compare the proteomic profile of the SD temporal cortex to our recently [34] measured SD dentate gyrus proteome. Major differences in atrophy levels between the two regions could complicate comparative analysis: loss of (a majority of) the neuronal cell type population could skew data and down-stream analysis. To address this potential complication, we analysed the raw proteomic data for the presence of major brain cell populations. Using the total set of measured peptides, we compared intensity-based absolute quantification (iBAQ) values as measure for absolute protein abundance for major brain cell types between SD patients and NDCs. Cell type-specific proteins were based on the sc/snRNAseq data sets used for cell type enrichment analysis, taking the top 50–200 proteins per cell type (Additional File 3). Additionally, the neuron-specific group of proteins was separately analysed in our MS-DAP pipeline to assess population normalization and differential abundancy in SD compared to NDC.

### Differential abundancy of TDP-43 and ANXA11 interactome proteins

As TDP-43 and ANXA11 are the major aggregating neuropathological proteins in SD, we aimed to determine the number of proteins with a role in the TDP-43 and ANXA11 interactome that were altered in SD patients. We extracted all known first order protein–protein interactors (PPI) of TDP-43 and ANXA11 from the STRING database [47] based on experimentally determined interactions, phylogenetic co-occurrence, and co-expression. The minimum required confidence score was set to 0.4 (medium confidence). Those proteins found to directly interact with TDP-43 and/or ANXA11 were checked for differential abundancy in both the SD temporal cortex and SD dentate gyrus proteome.

### Immunohistochemical analysis of post-mortem brain tissue

Immunostainings of candidate proteins in the temporal cortex were compared between a subset of four SD and two NDC samples, in addition to four TDP-43-negative neurodegenerative disease samples, namely two AD and two FTLD-TAU (Pick’s disease) samples, to assess specificity of the proteins for SD (Additional File 1). Formalin-fixed, paraffin-embedded tissue blocks were cut into 6-µm-thick sections. Deparaffinization was done in steps of gradually decreasing alcohol concentration. Antigen retrieval was performed in 0.01 M sodium citrate for 20 min in a pressure cooker. For CACNB4 staining, endogenous peroxidase was blocked using 0.6 H_2_O_2_ in phosphate-buffered saline (PBS; pH 7.0) for 30 min. After washing in PBS, 1.5% blocking serum from VECTASTAIN® Elite ABC-HRP Kit (Peroxidase [Rabbit IgG] PK-6101) in PBS was added. For HNRNPAB and RPS12 staining, blocking was done using 0.6% H_2_O_2_, 0.25% sodium azide in PBS and rinsing with PBS containing 0.5% protifar and 0.15% glycine. Tissue was incubated with primary antibody dilution (Table 1) at 4 °C overnight. After PBS rinsing, incubation with secondary antibody (Brightvision Poly-HRP-antiMs/Rb/Ra IgG one component, DPVO-HRP 55, Immunologic) was done for 1 h at room temperature, followed by another PBS rinse. For CACNB4 staining, avidin–biotin complex reagents from VECTASTAIN® Elite ABC-HRP Kit (Peroxidase [Rabbit IgG] PK-6101) were added for signal amplification according to manufacturer’s instructions. Sections were washed in PBS and incubated in DAB peroxidase substrate solution (D3939, Sigma-Aldrich) until desired stain intensity was achieved (2–15 min). Finally, after rinsing in demineralized water, hematoxylin counterstain was conducted and sections were mounted with Entellan. Images were made using the Olympus BX41 microscope (at 4X and 20X magnification) and cellSens Standard v2.3 software. Images were processed using QuPath v0.4.4 [4] and FIJI v.2.14.0 [43].

**Table 1:** Primary antibodies used for immunohistochemical validation

Candidate protein	Antibody	Company product	Dilution
CACNB4	CACNB4 polyclonal antibody	Protein Tech Group 17770–1-AP	1:200
HNRNPAB	hnRNP AB polyclonal antibody	Thermo Fisher Scientific PA5-27549	1:150
RPS12	RPS12 polyclonal antibody	Protein Tech Group 16490–1-AP	1:200

## Results

### Cohort description

Brain cortical tissues from the superior, middle or inferior temporal gyrus were collected from 18 SD patients with confirmed FTLD-TDP type C pathology and 25 NDCs (Additional File 1). NDC and SD groups did not differ significantly based on their age at death, sex, and CSF pH (data not shown). As expected in neurodegenerative disorders, the post-mortem brain weight was lower in SD patients (median = 945 g) than in NDCs (median = 1,231 g) (*p*-value = 2.59e-07). In addition, post-mortem duration (PMD) of SD patients (median = 325 min) was shorter than of NDCs (median = 475 min) (*p*-value = 0.0112).

### Proteomic signature of SD temporal cortex shows abundant differences

**Fig. 1:**
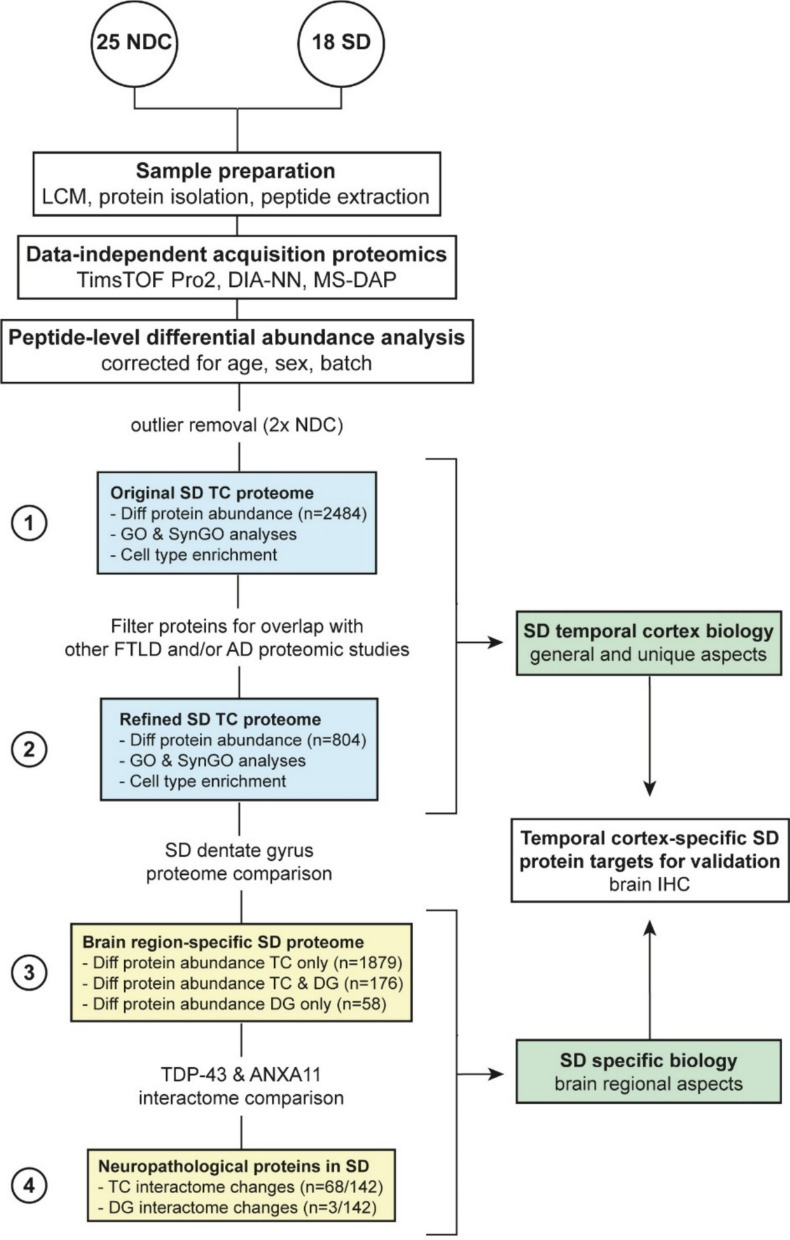
Schematic of the workflow and protein selection steps. Using published FTLD and AD proteomic literature we were able to refine our SD temporal cortex proteome towards SD unique aspects: proteins involved in common neurodegenerative processes or other FTLD disease subtypes were filtered out to enable focus on the SD disease process. Comparison to our recent SD dentate gyrus proteome enabled the study of brain-region specific aspects of the SD disease process, in which we also put additional emphasis on the role of neuropathological proteins TDP-43 and ANXA11. From these bioinformatic analysis we selected several SD temporal cortex-specific targets of interest for further validation using immunohistochemical analysis of post-mortem brain tissue. AD, Alzheimer’s disease; DG, dentate gyrus; Diff, differential; FTLD, frontotemporal lobar degeneration; GO, gene ontology; IHC, immunohistochemistry; LCM, laser capture microdissection; NDC, non-demented control; SD, semantic dementia; SynGO, synaptic gene ontology; TC, temporal cortex

To investigate quantitative proteomic changes, we used DIA LC–MS/MS proteomics on temporal cortical grey matter brain tissue. A schematic overview of the workflow and data analysis steps in this paper is presented in Fig. 1. In short, (1) we first assessed the proteomic signature of the temporal cortex in SD. To focus on protein change aspects specific to SD, (2) we refined the proteome by filtering out proteins that were shared with other FTLD subtypes and/or AD. To investigate brain region-specific protein changes in SD, (3) we compared the SD temporal cortex proteome to our previously generated SD dentate gyrus proteome. Lastly, (4) we specifically focussed on the role of neuropathological proteins TDP-43 and ANXA11 in both brain regions in SD. From these different detailed assessments, we selected several protein targets that appeared to be unique to the SD disease process in the temporal cortex region for validation study in a post-mortem neurodegenerative brain cohort.

**Fig. 2:**
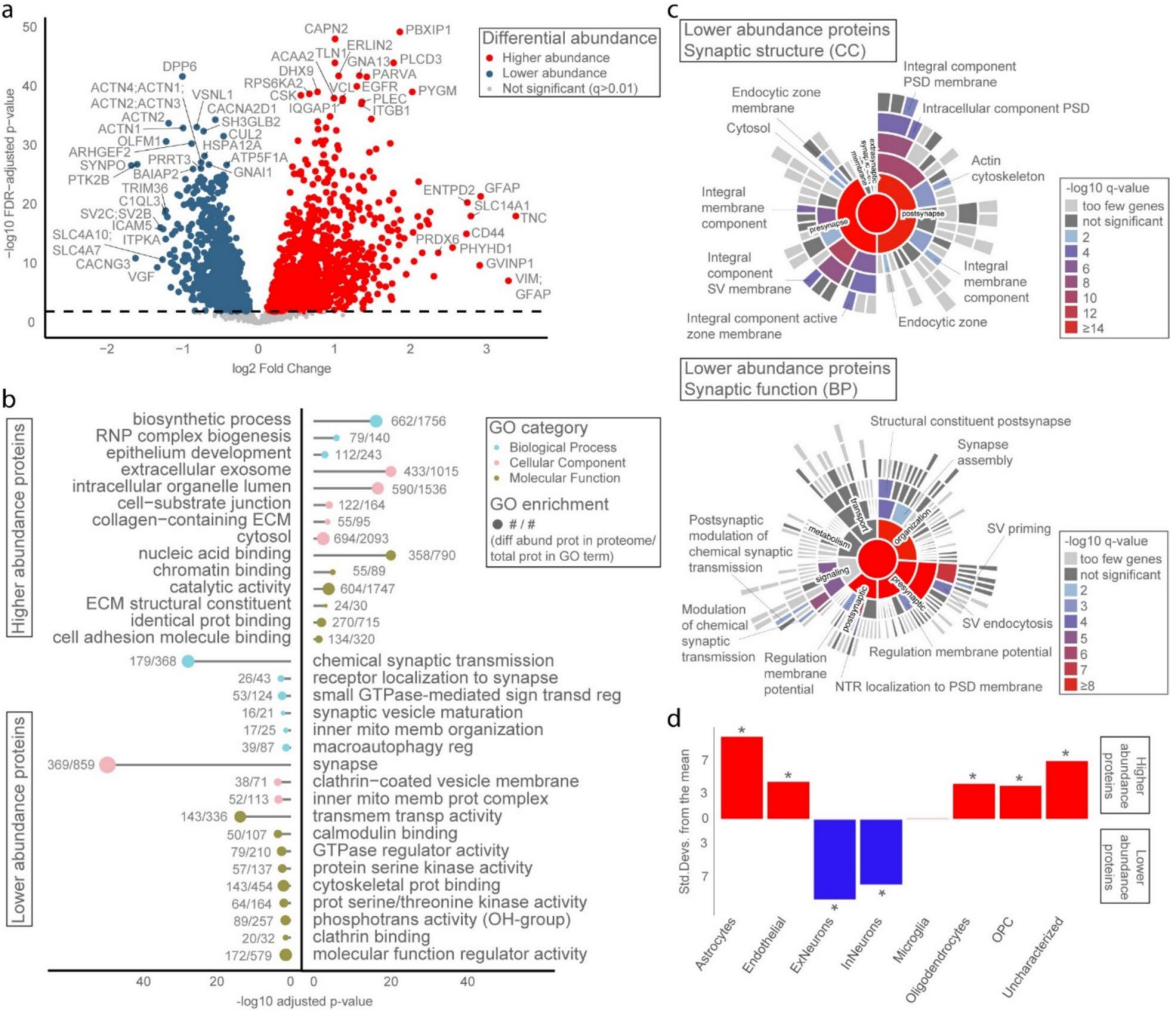
The temporal cortex in SD demonstrates major protein affecting all major brain cell types in the disease process. **a** Differential protein abundancy analysis in the temporal cortex demonstrated 2484 unique protein groups which are significantly different between SD and NDC (*q* < 0.01). Proteins with the highest significance and/or largest differential abundance are labelled. **b** GO term enrichment analysis on these 2484 proteins showed various DNA and mRNA-related terms, many synaptic processes, (synaptic) vesicles and vesicle transport, epithelial cell development, cell adhesion, and structural components of the ECM, the cytoskeleton, and mitochondria. **c** SynGO enrichment analysis on the 440 synaptic proteins with significantly lower abundance in the SD temporal cortex showed clear synaptic dysregulation across both pre- and postsynaptic compartments. **d** EWCE analysis on all significantly different proteins illustrated a role for almost all major brain cell types. BP, biological process; CC, cellular component; diff abund, differentially abundant; ECM, extracellular matrix; ExNeurons, excitatory neurons; FDR, false discovery rate; GO, gene ontology; InNeurons, inhibitory neurons; memb, membrane; mito, mitochondrial; NDCs, non-demented controls; NTR, neurotransmitter receptor; OPC, oligodendrocyte precursor cell; phosphotrans, phosphotransferase; prot, protein; PSD, postsynaptic density; reg, regulation; RNP, ribonucleoprotein; SD, semantic dementia; sign transd, signal transduction; Std. Devs., standard deviations; SV, synaptic vesicle; transmem, transmembrane; transp, transporter

In the temporal cortex SD proteome we detected abundances of 50,350 unique peptides. Quality analysis showed that two NDC samples were clear technical outliers with low peptide elution levels. After removal of these samples, filtering yielded 30,417 unique peptides, mapping to 4978 unique protein groups that were consistently measured across the 41 samples. We observed a median coefficient of variation of ~ 0.35 and ~ 0.25 in peptide and protein abundances, respectively, indicating high reproducibility between samples (Supplementary Fig. 1). The differential proteomic signature comprised 2484 unique protein groups in SD patients compared to NDCs (q-value < 0.01), of which 1376 were more abundant and 1108 were less abundant in SD versus NDC (Fig. 2a; Additional File 4). Proteins with the highest significance and/or largest differential abundance were labelled. This group of proteins (N = 51) indicates a variety of affected (neuronal) structures, i.e. the cytoskeleton, plasma membrane, dendrite, axon, and (post)synapse, and affected functions, i.e. cell adhesion, T cell-related immune processes, neurotransmitter receptor regulation, synapse organization, and synaptic signalling.

### GO analysis of the SD temporal cortex proteome highlights multiple biological processes involved in SD, including synaptic organization

GO overrepresentation analysis (Additional File 5) showed that proteins with higher abundance in SD are enriched for various DNA and mRNA processing GO terms, epithelial cell development, cell adhesion, and several terms related to the extracellular matrix (ECM). Proteins with lower abundance in SD are enriched for a large spectrum of synaptic processes, (synaptic) vesicles and vesicle transport, intracellular signalling, and structural components of the cytoskeleton and mitochondria. Significant driver GO terms (*q*-value < 0.05) are shown in Fig. 2b.

We zoomed in on synaptic biological processes by performing SynGO analysis (see Additional File 6). In total, 670 synaptic proteins were differentially abundant in SD temporal cortex compared to controls. Synaptic GO enrichment (*q*-value < 0.01) for synaptic proteins with lower abundance in SD (N = 440) pointed to involvement of both the pre- and post-synapse (Fig. 2c). In the presynapse, enriched terms were related to the endocytic zone and the regulation of its membrane potential, and to the priming and exo-/endocytosis of pre-synaptic vesicles. In the post-synapse, enriched terms highlighted the structural organization of the actin cytoskeleton and post-synaptic density membrane, and the regulation of membrane potential via neurotransmitter receptor localization.

### Cell type analysis reveals involvement of almost all major brain cell types in the SD temporal cortex proteome

To investigate which major brain cell type(s) are most involved and/or affected by the protein changes in SD, we performed EWCE analysis of significant differentially abundant proteins in the SD temporal cortex proteome (see Additional File 7). Cell type analysis (Fig. 2d) demonstrated significant enrichment (*q*-value < 0.05) for almost all major brain cell types. Protein with higher abundance in SD were enriched for astrocytes, endothelial cells, oligodendrocytes, and OPCs, whilst proteins with lower abundance in SD were enriched for both excitatory and inhibitory neuronal cell types.

### Proteins involved in RNA processing and regulation of presynaptic calcium levels appear to be specific to the SD disease fingerprint

To elucidate SD-specific disease fingerprints, we compared the SD temporal cortex proteome with published proteomic literature on neurodegenerative disorders in order to filter out general and/or non-specific protein features (see Additional File 2). First, we filtered out 98 protein groups not measured in any of the proteomic AD and FTLD studies. Then, 1519 protein groups that shared a differential signature with AD proteomes were filtered out, as they expectedly represent more common neurodegenerative disease features. Next, we filtered out 86 protein groups that shared a differential signature with FTLD-TAU proteomes, as they probably represent common FTD neurodegenerative features that are less related to unique SD pathways. Lastly, we filtered out 37 protein groups that shared a differential signature with FTLD-TDP proteomes, except for FTLD TDP type C proteomes. Though FTLD-TDP and SD patients share general TDP-43 pathology, we aimed to zoom in closely on SD-specific disease features. After this last step, the resulting refined SD temporal cortex proteome consisted of 3238 unique proteins groups (Additional File 4).

**Fig. 3:**
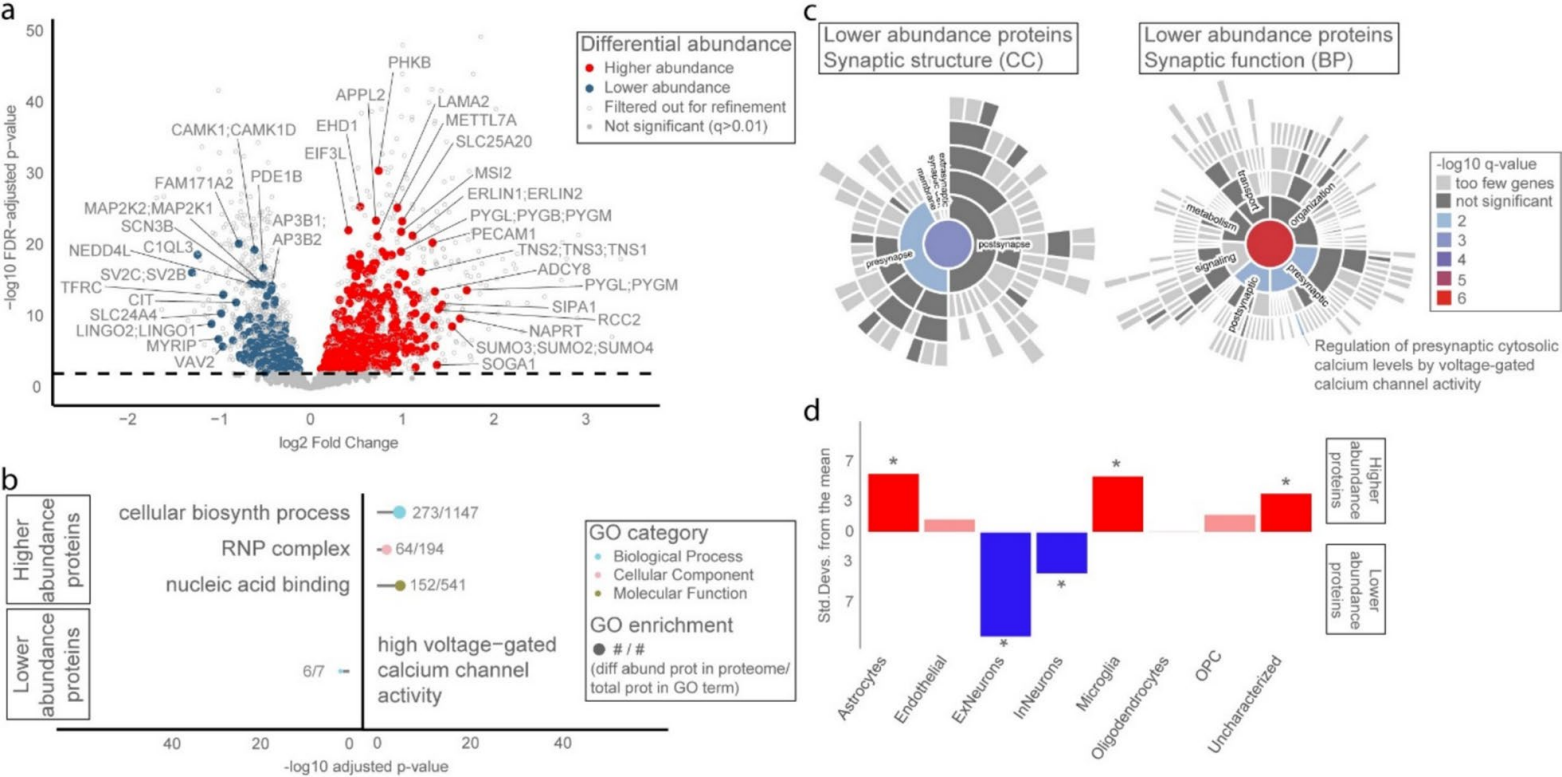
Refined SD temporal cortex proteome with corresponding functional enrichment and cell type enrichment analyses. Bioinformatic analysis of the refined SD temporal cortex proteome indicated a protein signature in which mRNA processing and pre-synaptic calcium homeostasis were more specific to the SD disease process. **a** Differential protein abundancy analysis in the refined temporal cortex proteome yielded 804 significantly different protein groups (*q* < 0.01). Proteins with the highest significance and/or largest differential abundance are labelled. **b** GO enrichment analysis on these 804 proteins showed that enrichment remains for driver terms related to the RNP complex, as well as emergence of a new enriched term related to calcium channel activity. **c** SynGO enrichment analysis on 172 synaptic proteins in the refined SD temporal cortex proteome specified this calcium channel activity further with emergence of the significant term voltage-gated calcium channel activity involved in regulation of presynaptic cytosolic calcium levels. **d** EWCE analysis after refinement suggested that endothelial cells, oligodendrocytes, and OPCs are more generally involved in neurodegeneration, while excitatory neurons and microglia could be more specifically affected and/or involved in the SD disease process. BP, biological process; CC, cellular component; biosynth, biosynthetic; diff abund, differentially abundant; FDR, false discovery rate; GO, gene ontology; OPC, oligodendrocyte precursor cells; prot, protein; Std. Devs., standard deviations

The refined SD temporal cortex proteomic signature comprised 804 differentially abundant proteins groups, of which 567 were more abundant and 237 were less abundant in SD compared to NDCs (*q*-value < 0.01) (Fig. 3a; Additional File 4). Proteins with the highest significance and/or largest differential abundance (N = 34) portrayed several aforementioned cellular processes, but also highlighted new processes like cell growth and migration, blood vessel maintenance and development, endosomal transport and autophagy, ion transport, glucose homeostasis, mRNA processing, and DNA repair. Interestingly, FAM171A2, a key regulator of progranulin (PGRN) expression [53], was one of the most significantly differentially abundant proteins in the refined SD temporal cortex proteome. Deficiency in PGRN levels is associated with development of neurodegenerative disease, including FTD subtypes with a mutation in C9ORF72 (TDP type A/B) [6] and in GRN (TDP type B) [3], and is linked to lysosomal dysfunction and TDP-43 aggregation [42]. While the exact dynamics between FAM171A2, PGRN and TDP-43 in SD remain to be investigated, our data suggest PGRN regulation also plays a role in SD.

The majority of previously enriched GO terms disappeared after re-analysis of the refined proteome, indicating that they represented shared features of neurodegenerative disease (Fig. 3b; Additional File 5). Two particular processes that appeared to be specific to the SD disease process point to the ribonucleoprotein (RNP) complex and the involvement of ion channel activity, the latter one being emphasized by emergence of novel enrichment for the term *high voltage-gated calcium channel activity* (q-value = 0.0498) in the GO analysis as well as *voltage-gated calcium channel activity involved in regulation of presynaptic cytosolic calcium levels* (q-value = 0.0013) in the SynGO analysis (Fig. 3c; Additional File 6). In the refined proteome, enrichment for endothelial cells, oligodendrocytes, and OPCs was lost (Fig. 3d; Additional File 7), indicating that most protein changes in these cells are depictive of a shared neurodegenerative disease process. Two cell types were enriched for proteins specific to the SD temporal cortex disease process: proteins with lower abundance in SD remained strongly enriched for excitatory neurons, even after filtering out shared neurodegenerative proteins, and microglia enrichment emerged for proteins with higher abundance in SD.

### Existence of a partially shared protein profile between temporal cortex and dentate gyrus in SD, but also temporal cortex-specific disease aspects

Recently [34], we performed proteomic analysis of the dentate gyrus of largely the same cohort of SD patients compared to NDCs. As these two regions greatly differ in neuropathological features in SD, a comparison between their proteomes would provide us with insights into region-specific disease changes. To address the possibility that differing atrophy levels in both regions interfere with comparative analysis, we examined the presence of cell type-specific protein populations in our data (Supplementary Fig. 2). Absolute protein abundancy values indicated that despite atrophy of the SD temporal cortex there were enough neurons left to enable meaningful comparison to the dentate gyrus proteome. For 3888 unique protein groups we had measurements in both data sets (Additional File 4) to compare proteomic changes between both brain regions.

**Fig. 4:**
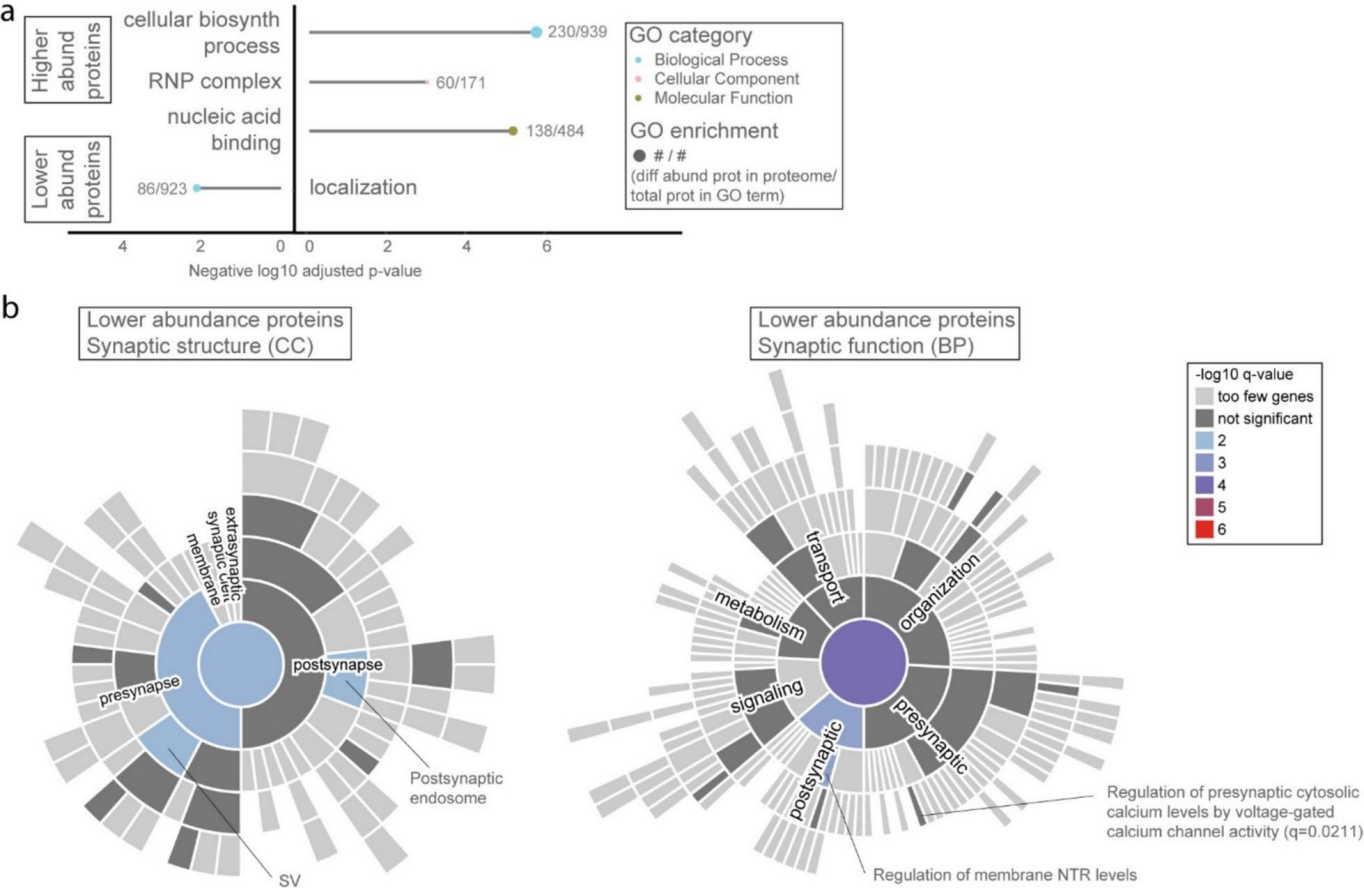
Proteins involved in the RNP complex and the regulation of presynaptic calcium levels potentially play a role in the SD disease process in the temporal cortex. **a** GO analysis on the proteins with higher abundance in SD temporal cortex highlights the RNP complex. **b** SynGO analysis on the proteins with lower abundance in SD temporal cortex demonstrates enrichment for the regulation of presynaptic calcium and postsynaptic NTR levels, the endosome, and SVs. abund, abundance; BP, biological process; biosynth, biosynthetic; CC, cellular component; GO, gene ontology; NTR, neurotransmitter receptor; RNP, ribonucleoprotein; SV, synaptic vesicle

While a set of 176 unique protein groups was differentially abundant in both regions in SD, the temporal cortex also harboured a large group of 1879 unique protein groups that was differentially abundant only in that region (*q* < 0.01, no fold change cut-off; Additional File 4). To focus on disease aspects distinct to SD, we applied filtering for FTLD and AD shared proteins here as well, resulting in 576 protein groups likely representing distinct aspects of the SD disease process in the temporal cortex (Additional File 4). These proteins showed a role for the RNP complex and mRNA processing on one hand, and for the presynaptic regulation of cytosolic calcium levels via voltage-gated calcium channel activity on the other hand, recapitulating these aspects as possibly specific to the temporal cortex region in SD (Fig. 4, Additional File 5 and 6). Supplemental analysis on the 176 unique protein groups shared between temporal cortex and dentate gyrus or the 58 unique proteins groups only differentially abundant in the dentate gyrus can be found in (Supplementary Fig. 3, Additional File 4 and 5).

### Striking regional differences in TDP-43 & ANXA11 interactome protein abundance in SD

**Fig. 5:**
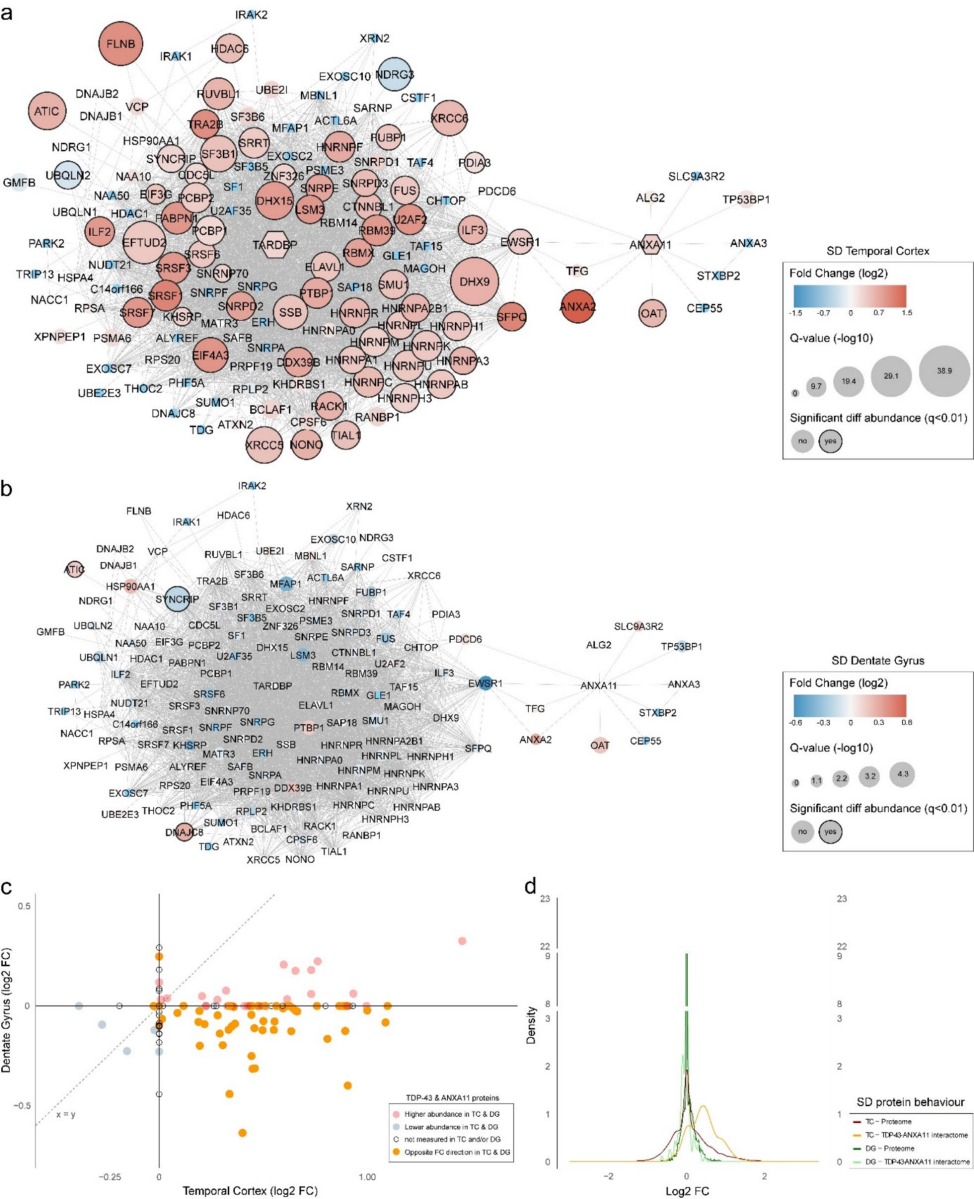
Protein–protein interaction network of the TDP-43-ANXA11 interactome demonstrated major changes in the temporal cortex, but little change in the dentate gyrus in SD. **a** Protein behaviour of the TDP-43 interactome (n = 131 interactor proteins) and ANXA11 interactome (n = 11 interactor proteins) in the SD temporal cortex. Here, 68 out of 101 measured interactome proteins (67.3%), including TDP-43 and ANXA11, were significantly differentially abundant in SD versus NDC, most of them showing higher abundance in SD. **b** Protein behaviour of the TDP-43-ANXA11 interactome in the SD dentate gyrus. In this brain region, only three out of 122 measured interactome proteins (2.5%) were differentially abundant. **c** Effect size plot of the log2 fold change of TDP-43-ANXA11 interactome proteins in the temporal cortex versus dentate gyrus in SD. The majority of proteins shows an opposite fold change direction between both regions (orange set). **d** Relative distribution of protein abundance changes in the entire proteome versus the TDP-43-ANXA11 proteome, for both the temporal cortex (red versus orange) and the dentate gyrus (dark green versus light green). Distribution of TDP-43-ANXA11 interactome protein fold changes in the temporal cortex clearly deviates from the other protein sets. Proteins marked with * were not measured in one or both SD datasets. Diff, differential; DG, dentate gyrus; FC, fold change; SD, semantic dementia; TC, temporal cortex

As mentioned, the temporal cortex and dentate gyrus differ in neuropathological features in SD. We investigated how exactly protein abundance within the interactome of neuropathological proteins TDP-43 and ANXA11 was affected in both brain regions in SD (Additional File 8 and Fig. 5). In the temporal cortex, two-thirds of proteins (67.3%) within the TDP-43-ANXA11 interactome showed significant differential abundancy in SD versus NDC (Fig. 5a). In the dentate gyrus, the interactome was significantly less affected, as only 3 proteins (2.5%) showed significant differential abundancy in SD versus NDC (Fig. 5b) Interestingly, neuropathological proteins TDP-43 and ANXA11 themselves were higher abundant in SD versus NDC in the temporal cortex, but abundance remained stable in SD versus NDC in the dentate gyrus. Furthermore, irrespective of statistical significance, a large set of interactome proteins demonstrates an opposing fold change direction in SD versus NDC when both brain regions are compared (Fig. 5c); while the majority of interactome proteins in the temporal cortex indicate higher abundancy, most of the interactome proteins in the dentate gyrus indicate lower abundancy. To prevent overinterpretation of these results, we performed an analysis of relative fold change distribution for the proteome versus the interactome (Fig. 5d). The unique pattern of distribution for interactome proteins in the temporal cortex (peak shift to positive fold changes) compared to general proteome changes in this brain region (peak centred around zero) indicates that higher protein abundancies in the TDP-43-ANXA11 interactome represent a biological occurrence in SD. Further comparison to distribution patterns for the dentate gyrus strongly suggests the TDP-43-ANXA11 interactome to be strikingly more affected in the temporal cortex than in the dentate gyrus in SD.

### Target protein validation using immunohistochemical analysis of post-mortem cortical SD brain tissue

From the different proteome assessments in this paper, we selected protein candidates that appeared to be unique to the SD disease process in the temporal cortex for validation in a post-mortem neurodegenerative brain cohort. We started with the set of 1879 unique proteins groups that had demonstrated significant differential abundance in the SD temporal cortex, but no significant difference in the SD dentate gyrus. From these, we focussed on proteins that were measured in the other FTLD and AD proteomes, but explicitly did not show any significant difference or did show a significant difference, but in opposite direction (n = 182). Next, we based candidate selection on their relation to the two possible SD-specific biological GO processes emerging from Fig. 4, e.g. the ‘RNP complex’ (GO) and ‘voltage-gated calcium channel activity involved in regulation of presynaptic cytosolic calcium levels’ (SynGO) (n = 104). In addition, candidates with well-measurable abundance differences (≥ ± 0.2 FC) were preferred (n = 97). From this pool of 97 candidate proteins, we decided to focus on targets with a proven synaptic expression (n = 35) and the commercial availability of a well-tested antibody (existence of either published immunohistochemical data or enhanced manufacturer validation). The final three selected candidates for immunohistochemical validation were CACNB4, HNRNPAB, and RPS12 (Table 2).

**Table 2:** Candidate selection for immunohistochemical validation

Candidate protein	Role in SD-specific GO biology	Differential abundance in SD TC (FC)	Differential abundance in AD proteomes	Differential abundance in FTLD-TDP proteomes	Differential abundance in FTLD-TAU proteomes
CACNB4	Presynaptic voltage-gated calcium channel activity (SynGO), presynaptic active zone/cytosol (SynGO)	Lower (0.796)	n.s	n.s	n.s
HNRNPAB	RNA binding (GO), macromolecule biosynthetic process (GO), cellular nitrogen compound biosynthetic process (GO), ribonucleoprotein complex (GO), cytosolic small ribosomal subunit (GO), translation at pre- and postsynapse (SynGO), pre- and postsynaptic ribosome (SynGO)	Higher (1.367)	Lower	n.s	n.s
RPS12	RNA binding (GO), macromolecule biosynthetic process (GO), cellular nitrogen compound biosynthetic process (GO), ribonucleoprotein complex (GO), presynaptic active zone/cytosol (SynGO)	Higher (1.514)	Lower	n.s	n.s

Candidates were selected for their apparent specificity for the SD disease process in the temporal cortex area, with no apparent role in the dentate gyrus. In addition, candidates showed an absence or different abundancy change in the published AD and FTLD proteomes. Furthermore, candidates were selected for their role in two possible SD-specific biological GO processes, relating to the RNP complex and calcium level regulation in the synapse. From the candidate pool, proteins with well-measurable abundance differences (≥ ± 0.2 FC) were preferred. Lastly, candidates were selected for their proven synaptic expression and depended on the availability of well-tested antibodies as well. AD, Alzheimer’s Disease; FC, fold change; FTLD-TAU, frontotemporal lobar degeneration with TAU pathology; FTLD-TDP, frontotemporal lobar degeneration with TDP-43 pathology; GO, gene ontology; n.s., not significant; SD, semantic dementia; SynGO, synaptic gene ontology; TC, temporal cortex.

**Fig. 6:**
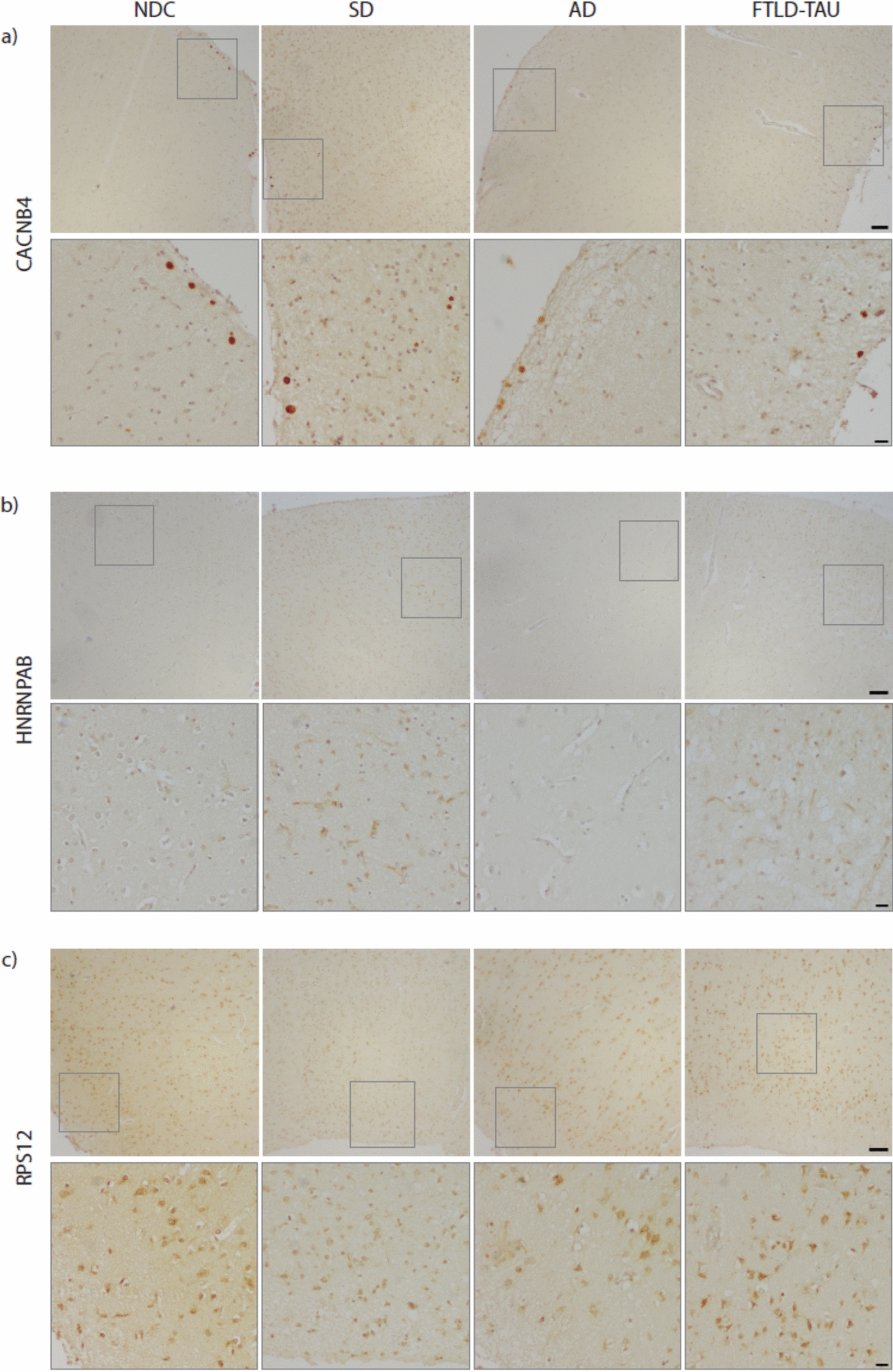
Immunohistochemical staining of CACNB4, HNRNPAB and RPS12 in a panel of neurodegenerative cases and NDCs. Protein expression was tested in temporal cortex tissue of NDC, SD, AD, and FTLD-TAU samples to validate the SD candidate targets. **a** CACNB4 staining is observed in extracellular vesicles in the upper cortical layers, especially on the surface, which are likely corpora amylacea. This staining is similar across all groups. **b** Anti-HNRNPAB antibody stains small blood vessel structures in the temporal cortex of SD and FTLD-TAU, but not in NDC or AD. **c** RPS12 positivity is seen across all cortical layers in NDC, SD, AD and FTLD-TAU, with more positivity in NDCs than in neurodegenerative subgroups. Upper panels are overview images, whilst bottom panels are magnified images taken from the inset location in the corresponding upper panel. Scale bar (overview) = 100 µm, scale bar (magnification) = 20 µm. AD, Alzheimer’s disease; FTLD-TAU, frontotemporal lobar degeneration with TAU pathology (here, two cases of Pick’s disease); NDC, non-demented control; SD, semantic dementia

Immunohistochemically, CACNB4 stained extracellular vesicles in the upper cortical layers, likely corpora amylacea (Fig. 6a). There was no evident cytoplasmic staining. While proteomic analysis found lower abundance for CACNB4 in SD versus NDC, visual assessment of IHC showed similar levels of CACNB4 positive staining across SD, NDC, AD and FTLD-TAU samples. HNRNPAB stained small blood vessel structures in the cortex of SD and FTLD-TAU, with staining intensity increasing with degree of cortical atrophy (Fig. 6b). There was no evident HNRNPAB positivity in NDCs, nor in AD. These IHC findings were similar to proteomic results, which showed that HNRNPAB was more abundant in SD than NDC. RPS12 stained neuronal cytoplasm in the cortical layers of NDCs and all three neurodegenerative subgroups. IHC showed equivalent-to-weaker RPS12 intensity in SD, AD and FTLD-TAU versus NDC (Fig. 6c). This is contrary to proteomic findings, in which RPS12 was found to be more abundant in SD than NDC. RPS12 positivity decreased with increasing cortical atrophy, irrespective of the neurodegenerative subgroup. Likewise, in specific cortical areas with less atrophy within neurodegenerative samples, more RPS12 positivity was observed (Fig. 6c, panel FTLD-TAU), stressing the inverted relationship between RPS12 positivity and degree of cortical atrophy.

## Discussion

This study describes the altered proteome of the temporal cortex in SD patients for the first time. Significant protein changes in the SD disease process point to involvement of affected RNA processing, cell adhesion, ECM organization, mitochondrial membranes, protein metabolism, and wide-spread dysfunction of synapses. All major brain cell types are influenced by these changes. We zoomed in on protein change aspects specific to SD, as these might resemble (early) key pathogenic disease mechanisms. Compared to other FTLD types and AD, two particular biological processes strongly emerged as possibly distinct for SD, i.e. mRNA processing by the RNP complex and the regulation of cytosolic calcium levels by voltage-gated channels specifically in the presynapse. Comparative analysis with our recently published SD dentate gyrus proteome established that these biological processes are partly region-specific to the temporal cortex in SD. In addition, this analysis demonstrated the existence of a partially shared protein profile between these differentially affected brain regions, but also of a major difference in the regulation of neuropathological TDP-43 and ANXA11 protein networks. As documented, the temporal cortex region in SD is heavily affected, while the dentate gyrus region is relatively spared, despite presence of neuropathological aggregates in both regions. Identification of these shared and region-specific proteome changes provides insight into the pathophysiological changes that are specific to the SD disease process and can help identify ideas for therapeutic strategies.

Our study highlighted a handful of biological processes in which proteins appeared to be specific to SD in the temporal cortex compared to other FTLD subtypes and AD, and compared to SD in the dentate gyrus. Most notable were the enrichment for mRNA processing and the RNP complex, and the regulation of cytosolic calcium levels by voltage-gated channels, specifically in the presynapse. TDP-43 is well-known for its in role in RNA processing [17] and disruption of these process is seen in all FTLD-TDP subtypes. Nonetheless, our results suggest that dysregulation of RNA processing in the temporal cortex in SD harbours unique aspects. It is possible that the unique form of TDP-43 type C neuropathology in SD holds the key to these disease-specific aspects of RNA dysregulation. Changes to several voltage-gated calcium channel proteins suggest impaired presynaptic calcium regulation is a feature of the SD disease process in the temporal cortex. This is in line with several studies demonstrating impaired calcium regulation as a result of cytoplasmic TDP-43 mis-localization in human TARDBP mutation cell culture models [11, 37]. However, the manner in which calcium impairments arise in SD might differ from other FTLD-TDP subtypes, resulting from lower abundance of calcium channel subunits instead of higher abundance as seen in these models. From these possible SD temporal cortex-specific biological processes, we selected several candidate proteins to validate their role in the SD disease process.

Validation of the synapse-expressed RNP complex protein HNRNPAB in our IHC post-mortem cohort is intriguing for our understanding of the SD disease process, while inconsistent results for RPS12 and CACNB4 staining potentially indicate they are more general markers of neuronal loss. The higher abundance of HNRNPAB in SD (and FTLD-TAU), with no changes in AD and NDC, was positively related to degree of cortical atrophy. Interestingly, HNRNPAB was mostly seen in blood vessels, possibly indicating the involvement of the neurovascular unit in the disease process. In future studies, it would be interesting to investigate the role of HNRNPAB in SD pathophysiology on a cellular level, and to corroborate that it is specific to TDP-43 subtype C pathology.

The existing contrast between the temporal cortex and dentate gyrus regions in SD warrants the question whether it reflects the existence of separate disease processes within both regions or should be attributed to different stages of the same disease process. Potentially, the dentate gyrus represents an ‘early’ stage of the SD disease trajectory which would hypothetically evolve into the diseased state of the temporal cortex upon an extended disease course. Comparison between our SD temporal cortex and dentate gyrus proteome datasets did not suggest these regions represent completely different disease profiles, as proteomes partially overlapped. A shared set of 176 differentially abundant proteins showed enrichment for the extracellular region, cell–cell junctions, and cell adhesion molecules, of which 21 proteins were distinct to SD. These SD-distinct proteins play a role in cell adhesion (including two cadherin-catenin complex proteins, CTNND2 and JUP), protein quality control, the stress response, and DNA processing. However, comparison between both regions also revealed sufficient differences to suggest that the DG proteome is not a pre-stage to the temporal cortex proteome. This is also supported by the profound difference in TDP-43 (and ANXA11) interactome protein abundances between both regions; while in the temporal cortex more than 60 percent of the interactome is altered in SD versus NDC, only 2.5 percent is altered in the dentate gyrus in SD versus NDC. Possibly, the disruption of TDP-43 (and ANXA11) in the temporal cortex in SD has more/more wide-spread pathogenic effects than in the dentate gyrus and other FTLD-TDP subtypes [40]. This year, a study demonstrated that a graded profile of atrophy exists along the hippocampal axis in SD, with anterior regions being more affected than posterior regions [27]. This suggests that a biological disease trajectory could be present along this axis as well. Detailed study of hippocampal subregions will help elucidate disease overlap with the temporal cortical regions further.

Another relevant aspect to mention in the context of proteome changes, is the importance of post-translational modifications (PTMs) to the proteome. PTMs change protein properties, increase the (functional) diversity of the proteome and play a key role in neurodegenerative disorders [15]. For the FTLD-TDP subtypes specifically, the role of TDP-43 (hyper-)phosphorylation is being studied extensively [16]. In our current study, we focussed on changes to the ‘basic’ proteome in SD and did not take into account the level of PTM changes, such as phosphorylation. To deepen our understanding of the SD disease process and to examine in detail the differences between the temporal cortex and dentate gyrus regions, especially in relation to the TDP-43 (and ANXA11) interactome, future phosphoproteomic analysis of our cohort is a promising approach.

As can be seen from the differential abundance analysis in Fig. 2a, the proteome in SD samples strongly differs from that in NDC samples, with approximately half of all proteins showing significant changes. This substantial difference might be (partially) caused by the loss of specific cell type populations (e.g. neurons and oligodendrocytes) within neurodegenerative tissue. As we ensure the loading of equal amounts of protein material for all samples, neurodegenerative cell type loss could result in overrepresentation of other cell types (e.g. glial cell types) still present in the tissue. Previously, we demonstrated that expected changes to cell populations in post-mortem tissue only partially explain proteomic changes in a genetic FTD cohort [32] and cell population-specific protein analysis for our SD temporal cortex cohort here demonstrated there are enough neurons left to ensure the relevance of our proteomic dataset.

The validation analysis of proteomic-guided candidate proteins demonstrated the difficulty of reproducing protein-level results using different methods: the more elementary approach to protein measurements of IHC could yield different results compared to the more sensitive peptide-based approach of quantitative proteomic analysis. Furthermore, IHC analysis on human post-mortem brain tissue has its shortcomings, dealing with variability of antigen preservation and staining ability of tissues [50]. In addition, asymmetry in brain atrophy in patients could lead to differing findings between left- and right-sided hemispheres. In this project, we were constrained by the protocol of the Netherlands Brain Bank, which freezes left-sided tissue (e.g. for proteomic analysis) and preserves right-sided tissue in paraffin-blocks (e.g. for IHC analysis). For future research it could be advantageous to use tissue of the same hemisphere for all experiments to ensure comparability. Lastly, disrupted tissue integrity and neuronal cell loss hinder quantification and cell-type specific analysis of protein target expression in IHC. Future (single) cell type-specific proteomic [5, 51] or spatial proteomic imaging [49] approaches will help elucidate the cell type specific role of proteome changes in SD.

## Conclusion

This study established the existence of SD-specific alterations in the proteome of the temporal cortex in SD. Disrupted (synaptic) mRNA metabolism and synaptic calcium regulation might be key processes that set this subtype apart from other FTLD-TDP subtypes. Comparison to our SD dentate gyrus proteome illustrated the presence of a partially shared profile, but also region-specific changes. Moreover, differences in TDP-43 and ANXA11 protein abundance between both regions corroborates the documented morphological difference in TDP-43 neuropathology between both regions. Further study of these shared and region-specific SD proteome profiles can provide insights into the pathophysiological changes that are specific to the SD disease process.

## Supplementary Information

Additional file1.

Additional file2.

Additional file3.

Additional file4.

Additional file5.

Additional file6.

Additional file7.

Additional file8.

**Supplementary Figure 1. Coefficient of variation analysis for peptide and protein abundances in SD versus NDC**. Analysis showed median COVs of ~0.35 and ~0.25 for peptide and protein abundances, respectively, indicating high reproducibility between samples

**Supplementary Figure 2. Analysis of the total set of measured proteins within the SD temporal cortex established the presence of a neuron-specific protein population, despite atrophy**. a) Distribution of iBAQ values as measure for absolute protein abundance across major brain cell types demonstrated that SD patients have a lower number of neuron-specific copy numbers in the temporal cortex than NDCs, while having a higher amount of glial copy numbers, especially for the astrocyte and microglia populations. b) Nonetheless, subsequent statistical analysis of neuron-specific proteins demonstrated a normally distributed population of differentially abundant proteins, with approximately half showing significantly different abundancies in SD compared to NDCs. As pure ‘overall loss’ of neuronal cells in SD would have resulted in an absence of differential expression in this type of subset analysis, we concluded that despite abundant atrophy there are enough neurons left in the temporal cortex of SD to enable meaningful bioinformatic analysis of the data. c) Distribution of major brain cell type iBAQ values in the dentate gyrus demonstrated that SD patients had comparable but less prominent changes as seen in the temporal cortex. d) Subsequent statistical analysis of neuron-specific proteins also demonstrated a normally distributed population of differentially abundant proteins, but almost no significant differential abundancy in SD compared to NDCs

**Supplementary Figure 3. Comparison between temporal cortex and dentate gyrus regions in SD reveals a partially shared proteome profile, as well as brainregion specific differences**. a) Effect size comparison of shared protein abundancies of the SD proteome between the temporal cortex and dentate gyrus is shown. Most of the shared proteins displayed fold changes in similar direction, but 16 had opposing fold change behaviour between both regions. Labelled proteins mark the 21 protein groups that were not differentially abundant in the FTLD/AD proteome literature and might be distinctly involved in the SD disease process in both brain regions. b) GO enrichment analysis on the 176 shared proteins showed enrichment for driver terms related to the extracellular region, cell-cell junctions, and cell adhesion molecules. c) Differential protein abundance for the 58 unique protein groups which are significantly different between SD and NDC only in the dentate gyrus. Proteins with the highest significance and/or largest differential abundance are labelled. d) GO enrichment analysis on these 58 proteins showed enrichment for driver term ‘extracellular space’. abund, abundance; AD, Alzheimer’s disease; DG, dentate gyrus; diff, differentially; FC, fold change; FDR, false detection rate; FTLD, frontotemporal lobe degeneration; GO, gene ontology; prot, protein; SD, semantic dementia; TC, temporal cortex

## Data Availability

The mass spectrometry proteomics data have been deposited in the ProteomeXchange Consortium via the PRIDE [37] partner repository with the dataset identifier PXD 062722. All data from downstream bioinformatics analysis and from immunohistochemical analysis experiments are included in this article and its additional files.

## Abbreviations

AD: Alzheimer’s disease
BP: Biological process
CC: Cellular component
DIA: Data-independent acquisition
ECM: Extracellular matrix
EWCE: Expression-weighted cell type enrichment analysis
FDR: False discovery rate
FTD: Frontotemporal dementia
FTLD: Frontotemporal lobar degeneration
FTLD-TAU: Frontotemporal lobar degeneration with TAU pathology
FTLD-TDP: Frontotemporal lobar degeneration with TDP-43 pathology
GO: Gene ontology
iBAQ: Intensity-based absolute quantification
MF: Molecular function
MS: Mass spectrometry
NDC: Non-demented control
PGRN: Progranulin
PPI: Protein–protein interactors
PTM: Post-translational modification
RNP: Ribonucleoprotein
scRNAseq: Single cell RNA sequencing
SD: Semantic dementia
snRNAseq: Single nuclei RNA sequencing
svPPA: Semantic variant of primary progressive aphasia
SynGO: Synaptic gene ontology
