# Correction: Proteomics of the temporal cortex in semantic dementia reveals brain-region specific molecular pathology and regulation of the TDP-43-ANXA11 interactome

**DOI:** 10.1186/s40478-025-02144-3

**Published:** 2025-10-24

**Authors:** Suzanne S. M. Miedema, Ana Rajicic, Merel O. Mol, Iryna Paliukhovich, Remco V. Klaassen, Renee van Buuren, Ka Wan Li, Frank T. W. Koopmans, Harro Seelaar, Jeroen G. J. van Rooij, August B. Smit, John C. van Swieten

**Affiliations:** 1 Department of Molecular and Cellular Neurobiology, Center for Neurogenomics and Cognitive Research, Amsterdam Neuroscience, Vrije Universiteit Amsterdam, Amsterdam, The Netherlands; 2 Department of Neurology and Alzheimer Center Erasmus MC, Erasmus University Medical Center, Rotterdam, The Netherlands; 3 Department of Molecular and Cellular Neurobiology, Center for Neurogenomics and Cognitive Research, Amsterdam Neuroscience, Vrije Universiteit Amsterdam, Remco V. Klaassen, Amsterdam, The Netherlands

**Correction to: Acta Neuropathologica Communications (2025) 13:162**

10.1186/s40478-025-02077-x

In this article [1], the term ‘immunoblotting’ was incorrect and should have read ‘immunohistochemical’.

The original article has been corrected.
