# Supplementary figures and images for: Proteomics of the temporal cortex in semantic dementia reveals brain-region specific molecular pathology and regulation of the TDP-43-ANXA11 interactome
Source: Acta Neuropathol Commun. 2025 Jul 25;13:162. doi: 10.1186/s40478-025-02077-x (PMC12291262; doi:10.1186/s40478-025-02077-x)

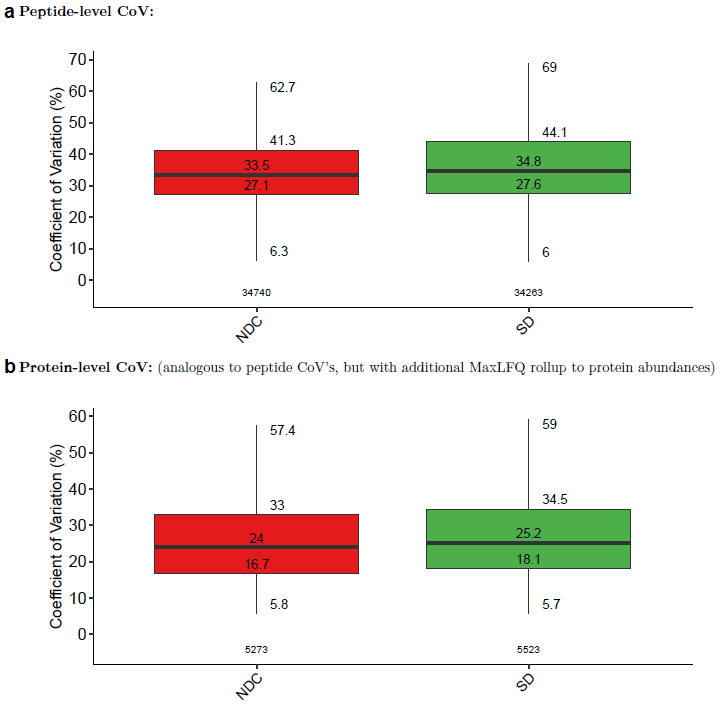

Supplement: Supplementary file 9 — Supplementary Figure 1. Coefficient of variation analysis for peptide and protein abundances in SD versus NDC. Analysis showed median COVs of ~0.35 and ~0.25 for peptide and protein abundances, respectively, indicating high reproducibility between samples [file 40478_2025_2077_MOESM9_ESM.tif]

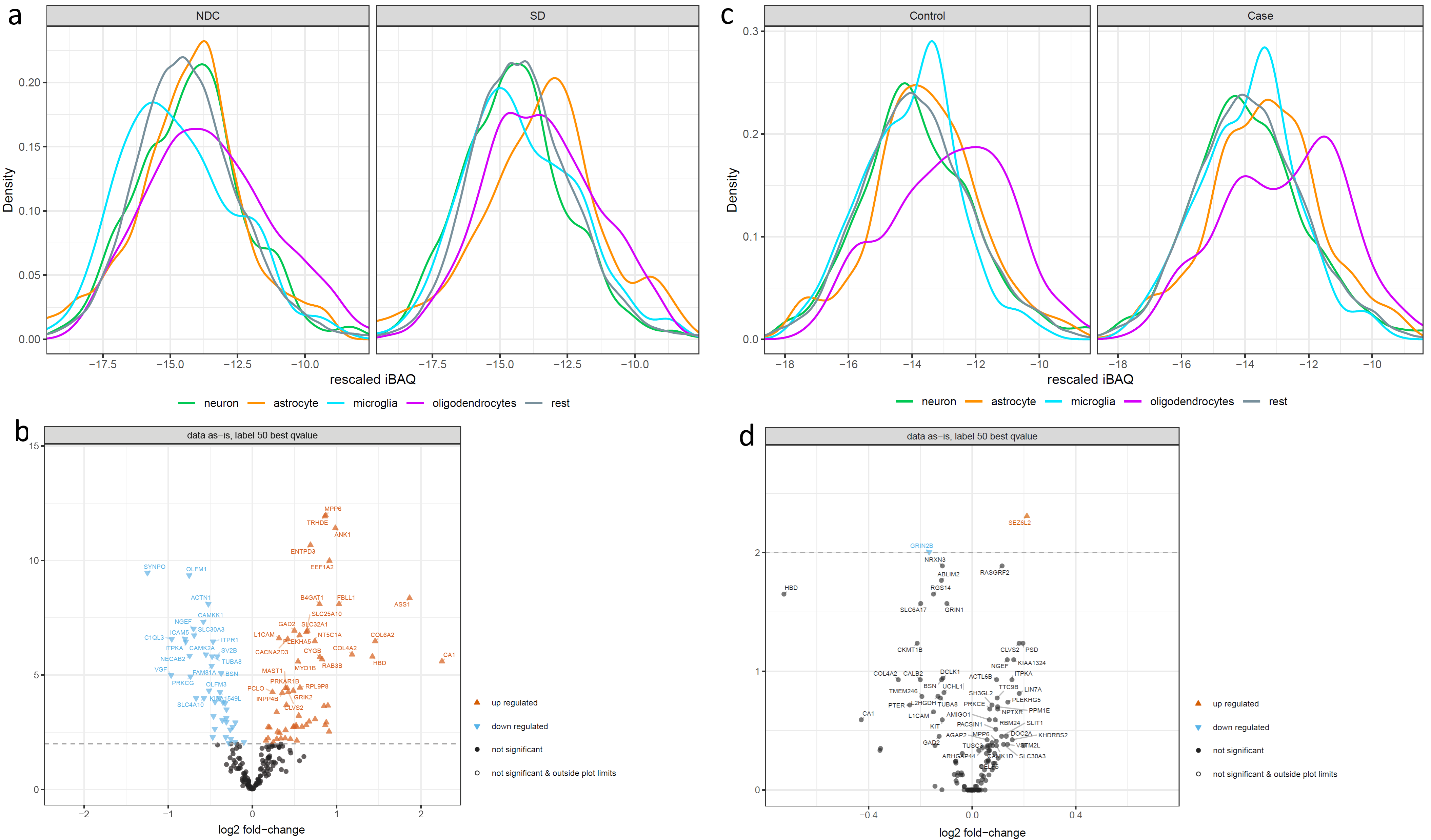

Supplement: Supplementary file 10 — Supplementary Figure 2. Analysis of the total set of measured proteins within the SD temporal cortex established the presence of a neuron-specific protein population, despite atrophy. a) Distribution of iBAQ values as measure for absolute protein abundance across major brain cell types demonstrated that SD patients have a lower number of neuron-specific copy numbers in the temporal cortex than NDCs, while having a higher amount of glial copy numbers, especially for the astrocyte and microglia populations. b) Nonetheless, subsequent statistical analysis of neuron-specific proteins demonstrated a normally distributed population of differentially abundant proteins, with approximately half showing significantly different abundancies in SD compared to NDCs. As pure ‘overall loss’ of neuronal cells in SD would have resulted in an absence of differential expression in this type of subset analysis, we concluded that despite abundant atrophy there are enough neurons left in the temporal cortex of SD to enable meaningful bioinformatic analysis of the data. c) Distribution of major brain cell type iBAQ values in the dentate gyrus demonstrated that SD patients had comparable but less prominent changes as seen in the temporal cortex. d) Subsequent statistical analysis of neuron-specific proteins also demonstrated a normally distributed population of differentially abundant proteins, but almost no significant differential abundancy in SD compared to NDCs [file 40478_2025_2077_MOESM10_ESM.png]

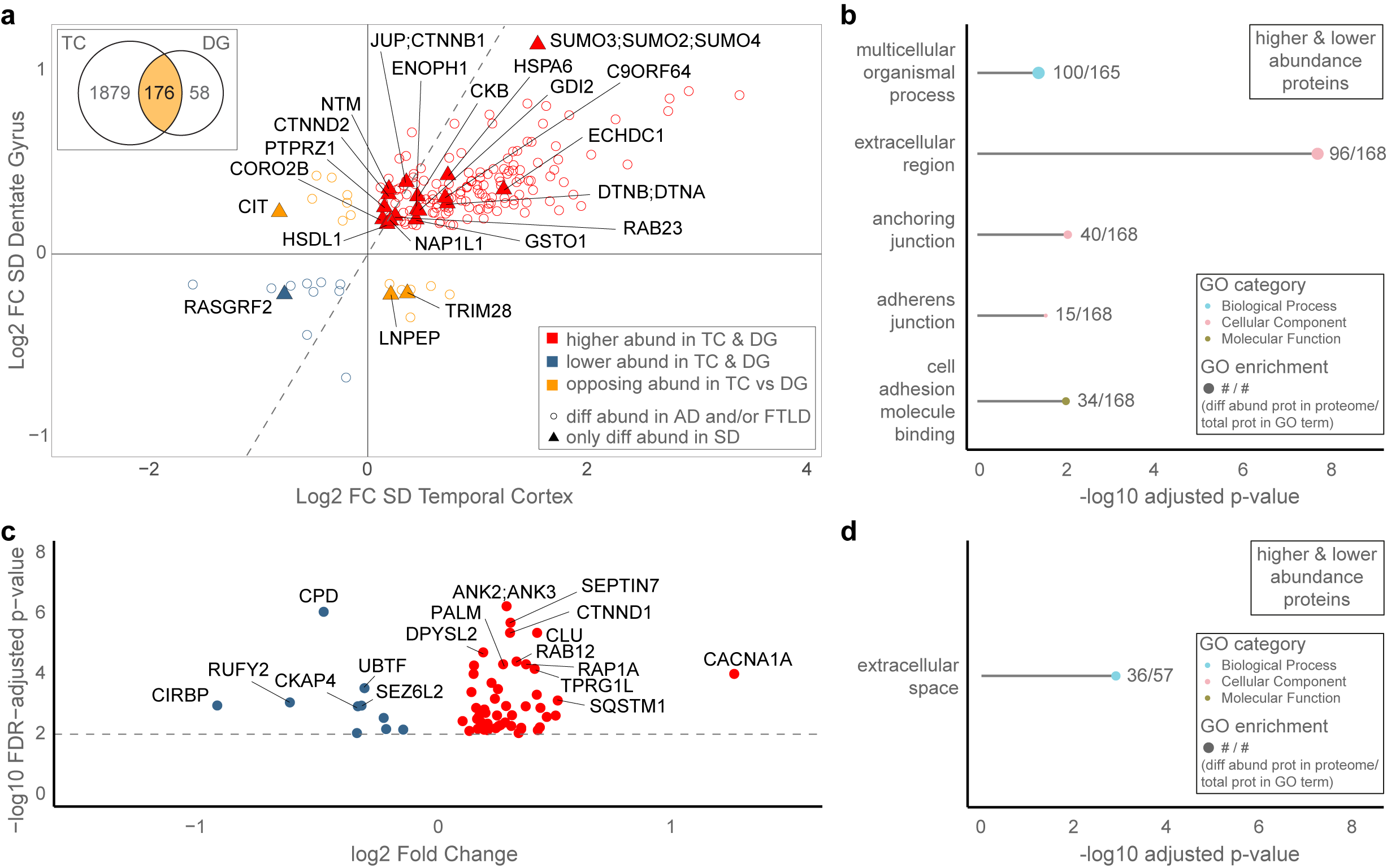

Supplement: Supplementary file 11 — Supplementary Figure 3. Comparison between temporal cortex and dentate gyrus regions in SD reveals a partially shared proteome profile, as well as brainregion specific differences. a) Effect size comparison of shared protein abundancies of the SD proteome between the temporal cortex and dentate gyrus is shown. Most of the shared proteins displayed fold changes in similar direction, but 16 had opposing fold change behaviour between both regions. Labelled proteins mark the 21 protein groups that were not differentially abundant in the FTLD/AD proteome literature and might be distinctly involved in the SD disease process in both brain regions. b) GO enrichment analysis on the 176 shared proteins showed enrichment for driver terms related to the extracellular region, cell-cell junctions, and cell adhesion molecules. c) Differential protein abundance for the 58 unique protein groups which are significantly different between SD and NDC only in the dentate gyrus. Proteins with the highest significance and/or largest differential abundance are labelled. d) GO enrichment analysis on these 58 proteins showed enrichment for driver term ‘extracellular space’. abund, abundance; AD, Alzheimer’s disease; DG, dentate gyrus; diff, differentially; FC, fold change; FDR, false detection rate; FTLD, frontotemporal lobe degeneration; GO, gene ontology; prot, protein; SD, semantic dementia; TC, temporal cortex [file 40478_2025_2077_MOESM11_ESM.tif]
